# Modeling the Kinetics of Integrin Receptor Binding to Hepatic Extracellular Matrix Proteins

**DOI:** 10.1038/s41598-017-12691-y

**Published:** 2017-09-29

**Authors:** Shanice V. Hudson, Christine E. Dolin, Lauren G. Poole, Veronica L. Massey, Daniel Wilkey, Juliane I. Beier, Michael L. Merchant, Hermann B. Frieboes, Gavin E. Arteel

**Affiliations:** 1 Department of Pharmacology and Toxicology, University of Louisville, Louisville, KY 40202 USA; 2 Department of Bioengineering, University of Louisville, Louisville, KY 40208 USA; 3 James Graham Brown Cancer Center, University of Louisville, Louisville, KY 40202 USA; 4 University of Louisville Alcohol Research Center, University of Louisville, Louisville, 40202 KY USA

## Abstract

The composition of the extracellular matrix (ECM) proteins and the expression of their cognate receptors dictate cell behavior and dynamics. In particular, the interactions of ECM proteins with integrin receptors are key mediators of these cellular processes, playing a crucial role in the progression of several diseases of the liver, including inflammation, fibrosis/cirrhosis and cancer. This study establishes a modeling approach combining computation and experiments to evaluate the kinetics of integrin receptor binding to hepatic ECM proteins. ECM ligand concentration was derived from LC-MS/MS quantification of the hepatic ECM from mice exposed to chronic carbon tetrachloride (CCl_4_); receptor density was derived from published literature. Mathematical models for ECM-integrin binding kinetics that were developed incorporate receptor divalence and an aggregation scheme to represent clustering. The computer simulations reproduced positive cooperativity in the receptor aggregation model when the aggregation equilibrium constant (K_a_) was positive and greater than K_eq_ for divalent complex formation. Importantly, the modeling projected an increase in integrin binding for several receptors for which signaling is known to be increased after CCl_4_ exposure in the liver. The proposed modeling approach may be of use to elucidate the kinetics of integrin receptor binding to ECM proteins for homeostatic and diseased livers.

## Introduction

The extracellular matrix (ECM) consists of a broad range of components that interact bi-directionally with neighboring cells to create a dynamic and responsive microenvironment which regulates cell signaling, recruitment, and tissue function. The ECM not only provides structure and support for the cells in a tissue, but also acts as a reservoir for growth factors and cytokines and as a signaling mechanism by which cells can intercommunicate with their environment^1^. Quantitative and qualitative changes to the ECM structure and superstructure can impact overall health of the organ and the organism. In particular, the hepatic ECM changes predominantly described in published literature occur in the context of hepatic fibrosis, which is characterized by robust scarring of the liver with collagen fibrils. However, hepatic ECM is significantly more diverse than collagen ECM. Recent studies further indicate that hepatic ECM content changes dynamically in response to acute stress and injury^2–4^. Moreover, changes to the hepatic ECM may foster an environment that is conducive to cancer and metastasis. Although the concept that hepatic ECM changes drive hepatic dysfunction under several conditions is well understood, the mechanisms by which these effects are mediated are not.

Integrins comprise a family of heterodimeric transmembrane glycoprotein receptors that facilitate key interactions between cells and the ECM^5–7^. The binding of ECM ligands to integrins mediates critical processes, including cell adhesion, migration, proliferation, differentiation, inflammation and apoptosis. Indeed, several of the hallmarks of liver diseases and cancer (e.g., altered proliferation, angiogenesis and apoptosis) are hypothesized to be mediated via changes in ECM:integrin signaling^8^. Based on this assumption, integrins have become important therapeutic targets for diseases of dysregulation, including various cancers, fibrosis, and immune dysfunction. However, few integrin-based therapies have been effective to prevent and/or treat these diseases. This limitation is partly due, to an incomplete understanding of the complexity of the changes to integrin signaling under dysregulated conditions.

**Figure 1:**
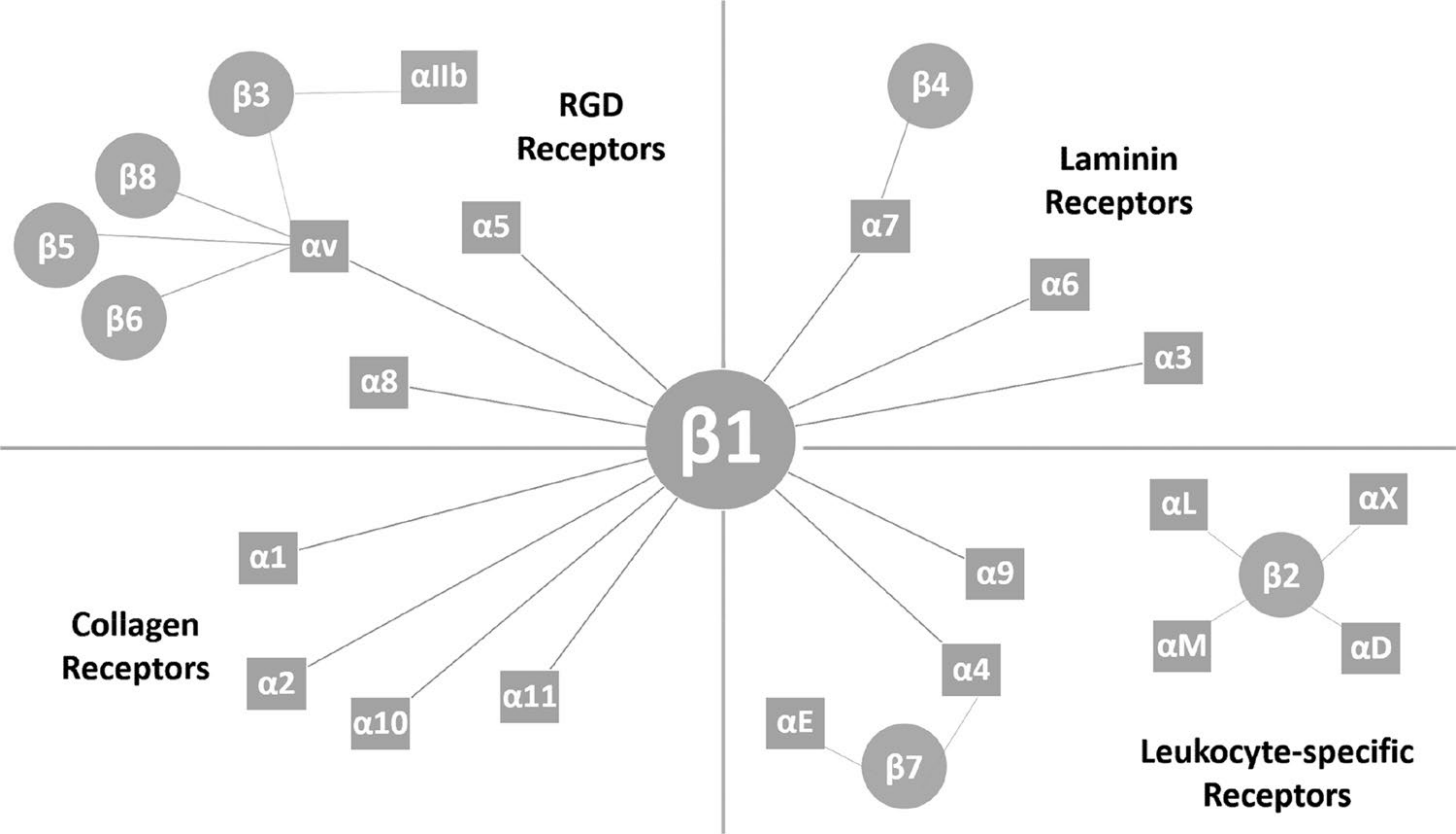
Repertoire of alpha and beta integrin subtype dimerization pairings. This diagram delineates the 24 possible integrin dimer species, classified by substrate type. For this study, of the integrins relevant to the CCl_4_ model, the collagen-binding β1 integrins and RGD-binding β1 and β3 receptors were evaluated. Binding here was treated as a two-step model with the α subunit binds ligand first, influencing ligand recognition and determinant of association kinetics. The β subunit binds second, creating bond stabilization and determining dissociation kinetics^14^, apropos to a divalent kinetics model with the α subunit being the high affinity site, and the β subunit as the low affinity site.

The kinetics of ECM:integrin interactions are highly intricate. Integrin receptor complexes are structured as non-covalently linked α and β subunits, the various combinations of which contribute to the diversity of receptor types^9^ (Fig. 1). The overall rate of binding is not driven simply by ligand binding to the receptor, but also by clustering at focal adhesion points and an increase in avidity for binding additional ligand (i.e., positive cooperativity). Masson-Gandais *et al*. described a two-step model wherein the α subunit binds ligand first, influencing ligand recognition and determinant of association kinetics^10^. The β subunit binds second, which creates bond stabilization and determines dissociation kinetics. Ligand binding to the extracellular domain activates the receptor and initiates its conformational changes to a high-affinity state^11,12^. This two-step process reflects a divalent kinetics model with the α subunit as the high affinity site, and the β subunit as the low affinity site^13^. In addition to binding processivity of individual receptors, ligand binding to distinct integrins favors subsequent binding by other receptors (i.e. focal adhesion clustering). Furthermore, integrin receptors bind promiscuously to various ECM ligands, creating redundancy, competition and diversity in biofunctionality^5,9,14^. These complex interdependent factors affect the kinetics of ECM-integrin interactions in the intact organism. Promiscuity among the repertoire of ECM ligands and integrin receptors, particularly those with RGD-binding motifs, implies a differential pattern of binding relative to the amounts of substrate available^15,16^.

To explore these interactions as a system, several mathematical descriptions of integrin binding have been reported with outputs related to spatial clustering and signal transduction, liver fibrosis and haptotaxis^17–19^. Although these models recapitulate certain aspects of ECM-integrin interactions, they typically focus on one ligand (e.g. collagen or fibronectin) as the ECM substrate. In this study, modeling of integrin receptor binding kinetics is presented that considers divalent receptor characteristics and employs a simple model of integrin clustering. The kinetic indices of each integrin for each of its ligands were initially determined to establish a single-species integrin profile. Proteomic data were compiled that assess the liver ECM under homeostatic conditions as well as experimental fibrosis. These proteomic analyses provided information on relative abundance of hepatic ECM components to calibrate substrate concentrations for the kinetic simulations. Although data from human fibrotic livers has recently been analyzed^20^, an animal model was chosen here as it provides a more controlled environment for initial model calibration and testing. Longer term, by testing homeostatic conditions against the experimental treatment models, how the integrin binding phenotype changes in response to injury could be determined and used to predict the ECM-integrin binding within the context of transitional tissue remodeling.

## Results

**Figure 2:**
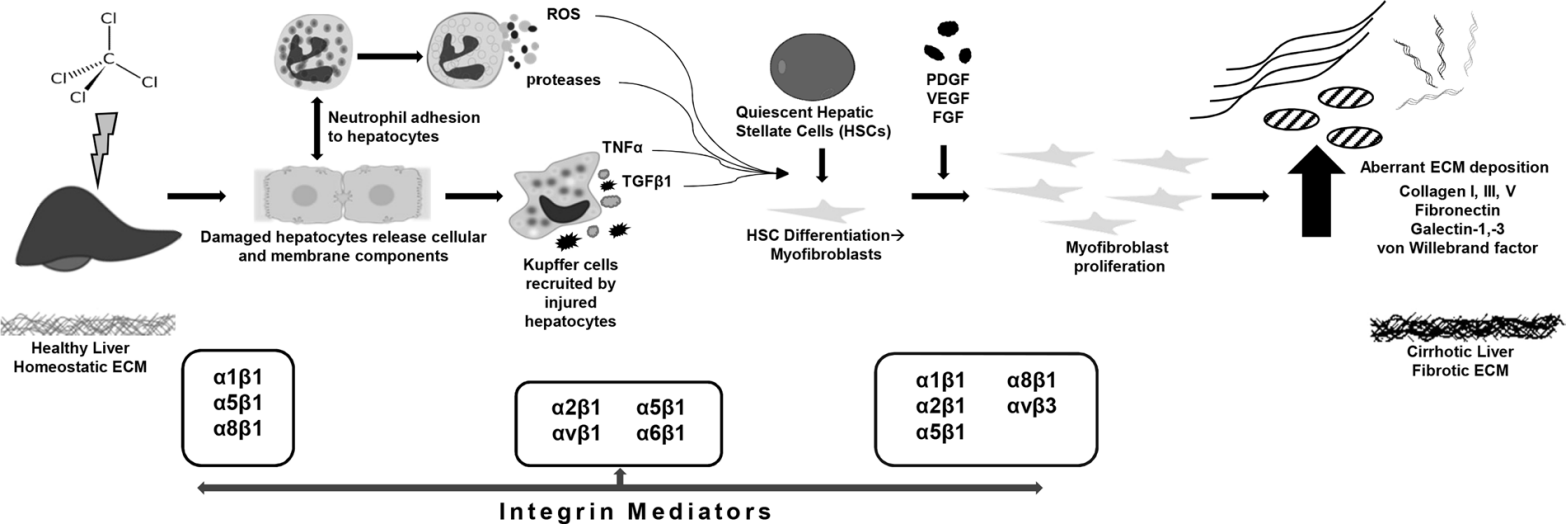
Schematic of aberrant ECM accumulation following CCl_4_ injury. Key extracellular matrix proteins (ECMPs) and cognate integrin receptors in CCl_4_ exposure mouse model of fibrosis. The phenomena include quiescent hepatic stellate cell (HSC) activation and their subsequent differentiation into myofibroblasts after which growth factor-induced proliferation leads to the aberrant ECM deposition that characterizes cirrhotic liver fibrosis. The chronic inflammatory response involves impaired matrix degradation which further contributes to dyshomeostasis of ECM proteins, and therefore tissue structure and errant signal transduction. Following exposure to CCl_4_, damaged hepatocytes release cellular and membrane components^8^, leading to recruitment of neutrophils and Kupffer cells. Profibrogenic and proinflammatory cytokines, reactive oxygen species (ROS), and proteases are released from resident immune cells, leading to stimulation and activation of quiescent HSCs, inducing their differentiation to myofibroblasts. Proliferation of activated myofibroblasts in response to fibrogenic factors results in excessive ECM deposition, leading to fibrotic scarring and end-stage liver disease. Integrin mediators known to be active in fibrotic pathology include β1, α1, α5, and α6 on hepatocytes, which correlate clinically with stage of fibrosis^8^. αvβ3 integrin signaling from HSCs/myofibroblasts is involved with regulating ECM-fibrolytic matrix metalloproteinases. De novo α8β1 expression in activated HSCs occurs in response to CCl_4_ injury; likewise, α1, α2, and α5 on HSCs is indicative of activation, enhancing attachment to basement membrane proteins^8^. Feed forward mechanism results from the fibrillar ECM itself enhancing HSC activation, implicating integrins α_1_β_1_, α_2_β_1_, and α_V_β_1_^33^.

As expected, 4 weeks of CCl_4_ exposure caused robust fibrotic scarring of the liver in our mouse model. The resultant phenotype of injury and fibrosis has been previously described to include degradation of basement membrane-like ECM and replacement with fibrillar collagens and other integrin ligands (Fig. 2)^21^. The canonical change in ECM content during hepatic fibrosis is an increase in collagen 1 deposition. However, as has been previously described^22,23^, several other proteins increase in response to CCl_4_-induced fibrosis.

Analysis of the proteomic data (Table 1) revealed ECM protein expression profiles, and a simple conversion for relating quantitative exponentially modified protein abundance index (emPAI) values to protein mass was employed as a proteomic ruler to estimate protein concentration under homeostatic and experimental treatment conditions^24,25^. Weighting the values with the concentration of extraction fractions, we estimated a relative protein concentration for ECM components. The composition of the liver ECM as quantitated via proteomic analysis has influence on integrin expression of cells that haptotactically migrate towards ECM protein gradients, and provides the pool of available ligands for subsequent binding.

**Table 1:** Quantitative Differential Protein Expression of Liver ECM following 4 Weeks of CCl_4_ Exposure.

Protein	GO Accession	MW (kDa)	Protein Abundance (µM)		Log_2_ FC
			Con	CCl_4_	
Col 1α1	CO1A1	138	3.60	6.42	0.83
Col 1α2	CO1A2	130	2.55	4.25	0.74
Col 3α1	CO3A1	139	0.75	1.64	1.13
Col 4α1	CO4A1	161	0.20	0.23	−0.24
Col 4α2	CO4A2	167	0.46	0.28	−0.71
Col 5α1	CO5A1	184	0	0.05	11.80
Col 5α3	Q9JLI2	172	0	0.06	11.90
Col 18α1	E9QPX1	182	0.07	0	−11.81
Dermatopontin	DERM	24	0	7.11	16.06
Fibronectin	FINC	273	0.18	0.47	1.40
Fibrinogen β chain	FIBB	55	3.13	0.56	−2.48
Fibrinogen γ chain	FIBG	49	5.94	1.94	−0.45
Galectin-1	LEG1	15	8.45	21.18	1.33
Galectin-3	LG3BP	64	0	0.63	13.97
von Willebrand factor A	VMA5A	87	0.33	0.84	1.33

Proteomic data for integrin-binding ECM proteins of interest (full dataset not shown). Zeroes were set to 0.00001 for calculation of Log_2_ fold change. The exponentially modified protein abundance index (emPAI) was used for estimation of absolute protein abundance^25^ and to approximate protein concentration of relevant integrin-binding proteins. Multidimensional protein identification technology (MudPIT) was used to artificially recombine fraction data from Mascot and SequestHT searches and produce quantitation that relates total protein signal in each treatment group^46,47^. To normalize for tissue fractionation, the dimensionless emPAI score was weighted with the concentration loaded for each fraction, i.e., 0.25 µg/µL, to calculate relative protein concentration as initial parameter values for the simulations.

**Figure 3:**
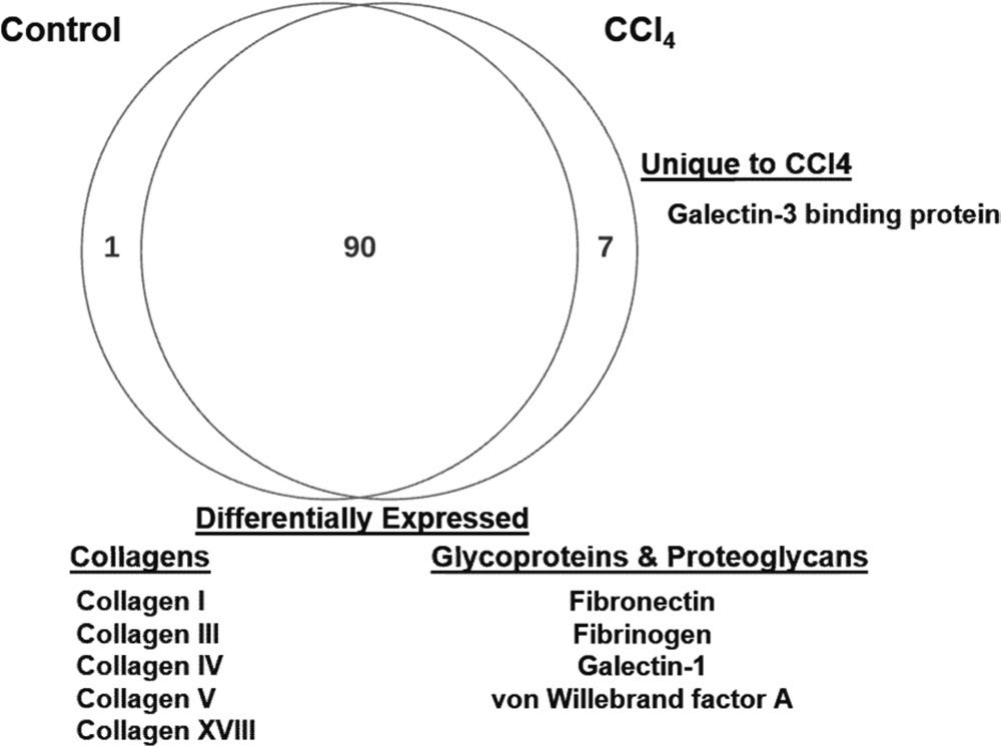
Qualitative Venn diagram of proteomic data. Differentially expressed proteins of interest for evaluation of ECMP-integrin bindings include collagens, fibrillar proteins, glycoproteins and proteoglycans. Of seven proteins uniquely expressed in the CCl_4_ experimental model, one ECMP protein, Galectin-3, was identified. One protein was unique to the control, and 90 were differentially expressed. ECMPs used for these simulations are listed in Table 1. Data for all relevant ECM proteins are given in Supplemental S1 Table.

Qualitatively, the majority of proteins identified were found in both the control and treatment groups; with seven proteins uniquely expressed in the CCl_4_ group and only one unique to the control group (Fig. 3). Collagens, glycoproteins and proteoglycans identified via proteomic analysis as ECM substrate were quantified and their relative concentration was determined (Table 1). Beta-1 and Beta-3 integrins selected for the simulations reflect those involved in hepatic events that relate to CCl_4_ fibrosis. Integrin-ECM binding microrates have been determined for various cell types and conditions.

**Figure 4:**
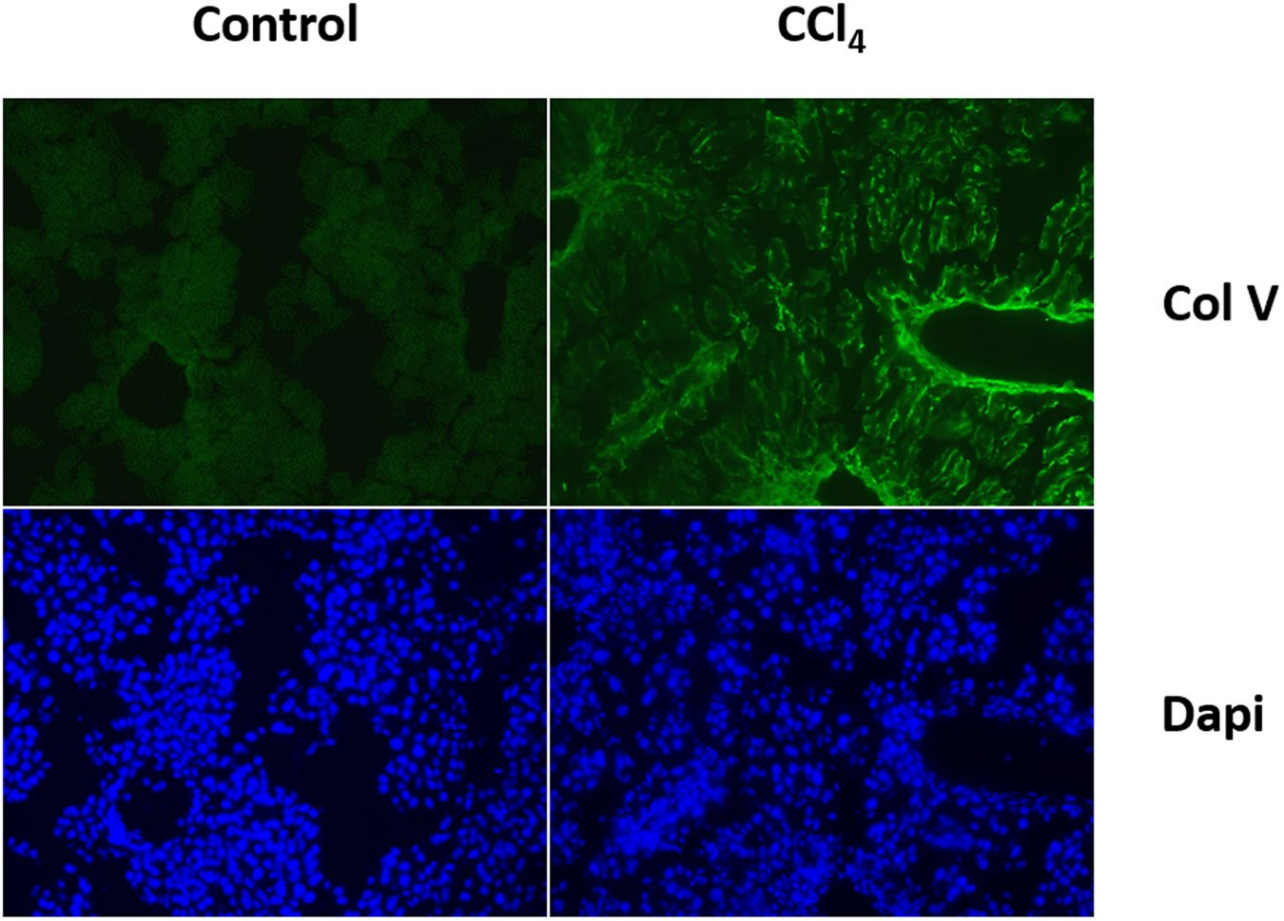
Immunofluorescent staining of hepatic cryosections. OCT sections were probed with Col V primary antibody and resolved with Alexa 488-tagged secondary antibody. The CCl_4_ treatment group shows a marked increase in Col V staining relative to the control.

Proteomic results were previously validated to confirm relative abundance of identified proteins qualitatively and quantitatively^2^. In particular, the amounts and distribution of collagens in the treatment group relative to the control were verified. Here, the presence of trace amounts of Col V in the CCl_4_ treatment group was validated to explore whether changes on the nanomolar scale would have pathological consequence. (Fig. 4).

Next, to provide for the capability of a system-level analysis, a computational framework was established using proteomic data for binding species to enable evaluation of integrin receptor binding kinetics (see modeling and experimental details in Methods). The model was developed taking into consideration sequential binding of subunits. *In silico* simulations of this model were parameterized using rate constants that correlate with published literature on binding, or otherwise estimated. The rates are listed in Tables 2 and 3.

**Table 2:** Binding Rate Parameters.

Integrin Species	Initial Value (nM)	Binding Kinetic Parameters (k_on_[s^−1^M^−1^];k_off_[s^−1^])							
		Col I^48,49^		Col IV^49^		Fibronectin		von Willebrand factor A^50^	
		k_on_	k_off_	k_on_	k_off_	k_on_	k_off_	k_on_	k_off_
α1β1^48,49,51^	0.0001	5.6 × 10^4^	1.3 × 10^−3^	8.0 × 10^5^	5.0 × 10^−3^	---	---	---	---
αVβ3	0.05	---	---	---	---	1.6 × 10^8^	3.5 × 10^−1^	1.6 × 10^4^	2.3 × 10^−2^

Initial conditions and reaction rate parameters of kinetic models – collagens, glycoproteins and proteoglycans. Rates for binding ECM components relevant to the CCl_4_ model are delineated per cognate integrin receptor. From published literature initial values of integrin receptor species are derived and given in nanomolar; similarly, binding rate parameters are given in s^−1^M^−1^ for k_on_ and s^−1^ for k_off_.

**Table 3:** Equilibrium binding constants for receptor aggregation model.

K_d_ (nM)	Reaction	Microrates (on; nM^−1^s^−1^/off; s^−1^)	Manipulations
K_i_	Integrin complex formation	*k*_2_ */k*_1_	K_p_ > K_i_
			increase in ligand affinity after aggregation
K_c_	Filling divalent unpaired receptor	*k*_4_ */k*_3_	K_c_ = 0.01 K_i_ decreased unpaired receptor K_eq_ for binding 2^nd^ ligand
K_a_	Empty receptor pairing with bound receptor	*k*_6_ */k*_5_	K_a_ > 0
			aggregation constant drives positive cooperativity
K_p_	Population of empty paired receptors	*k*_10_ */k*_9_	K_p_ > K_i_
			increase in ligand affinity after aggregation
			K_p_ = 100 K_x_ decreased aggregate receptor K_eq_ for binding 2^nd^ ligand
K_x_	Receptor saturation	*k*_8_ */k*_7_	K_p_ = 100 K_x_ decreased aggregate receptor K_eq_ for binding 2^nd^ ligand

Microrate parameters are derived from published values and set to implement positive cooperativity for sequential ligand binding and receptor aggregation. The rates for integrin complex formation (K_i_) are set to simulate an increase in ligand affinity post-aggregation. Populating an empty unpaired receptor is set with a hundredth fold decrease in K_eq_ for binding the second ligand. The aggregation equilibrium constant is set at ten times the equilibrium constant for initial complex formation to allow for aggregation to drive positive cooperativity. The population of empty paired receptors dictates an increase in ligand affinity after aggregation and is set to decrease aggregate receptor K_eq_ for binding second ligand for receptor saturation. These parameters are adapted from Wanant *et al*.^28^, and applied here to simulate positive cooperativity in receptor aggregation pairing so that the model can be initialized and implemented with proteomic data to evaluate binding profiles.

**Figure 5:**
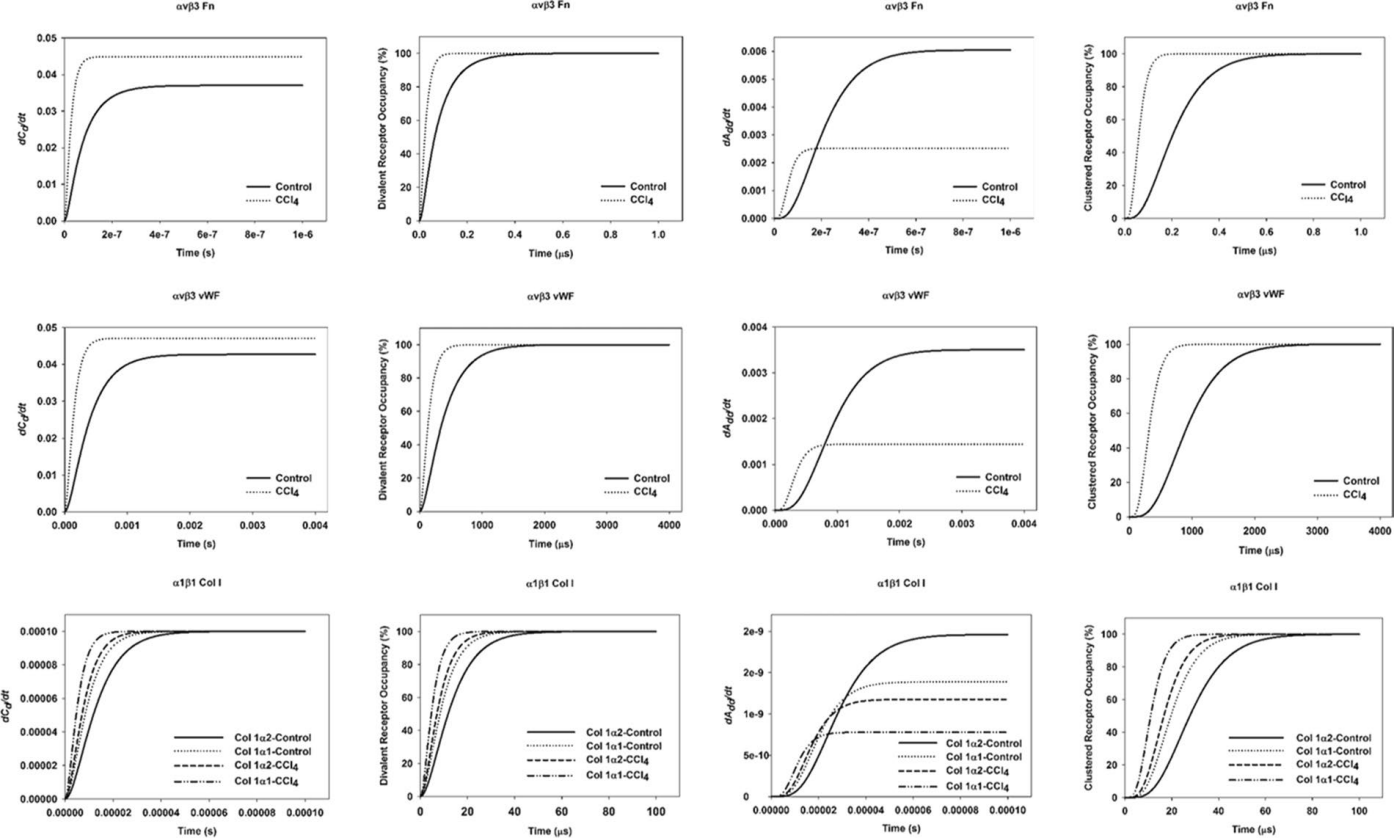
Kinetic simulations data. Model was initialized using ligand concentrations from proteomic analysis (Table 1) and kinetic rates listed in Table 2. The ECM:integrin binding pairs fibronectin:αvβ3, von Willebrand factor: αvβ3, and collagen I:α1β1 are shown, with binding curves and percent occupancy for fully occupied single divalent receptors (*C*_*d*_) and aggregated receptor pairs (*A*_*dd*_).

The simulations were initialized using binding constants from published literature; where values were not available, parameters were estimated accordingly (see Methods). Collagen fragments for collagen I and IV were plotted together (Fig. 5) and assumed to have the same rates of binding for the purposes of these experiments. For the other fragmented protein, fibrinogen, only the gamma subunit was considered due to the binding motif located within this fragment^26,27^. The binding microrates were set to recapitulate positive cooperativity in divalent receptor saturation and in receptor aggregation pairs, as stipulated in Wanant *et al*.^28^, wherein the aggregation equilibrium constant, K_a_, drove cooperativity in the aggregate model (Table 3).

The simulation graphs in Fig. 5 show left-shifted curves with increased ECM ligand abundance, indicating increased affinity and avidity for ligand. This is reflected in both the curves for fully occupied divalent receptors (*C*_*d*_) and for fully occupied aggregate receptor pairs (*A*_*dd*_). Steady state values (SS) were recorded for each simulation (Table 4).

**Table 4:** Steady state Values for Simulations of Binding.

ECMP, Treatment	Integrin Receptor	Steady state [*C*_*d*_]
vWF, CCl_4_	αvβ3	0.0471
vWF, Control	αvβ3	0.0427
Fibronectin, CCl_4_	αvβ3	0.0448
Fibronectin, Control	αvβ3	0.0369
Col 1α1, CCl_4_	α1β1	0.0001
Col 1α2, CCl_4_	α1β1	0.0001
Col 1α1, Control	α1β1	9.99 × 10^−5^
Col 1α2, Control	α1β1	9.99 × 10^−5^
**ECMP**, **Treatment**	**Integrin Receptor**	**Steady state [** ***A***_***dd***_ **]**
vWF, CCl_4_	αvβ3	0.0014
vWF, Control	αvβ3	0.0035
Fibronectin, CCl_4_	αvβ3	0.002
Fibronectin, Control	αvβ3	0.006
Col 1α1, CCl_4_	α1β1	7.79 × 10^−10^
Col 1α2, CCl_4_	α1β1	1.18 × 10^−9^
Col 1α1, Control	α1β1	1.39 × 10^−9^
Col 1α2, Control	α1β1	1.96 × 10^−9^

Steady state Values for Simulations of Binding. Steady state (SS) values for a fully occupied single divalent receptor (*C*_*d*_) and saturated aggregated receptor pairs (*A*_*dd*_). Top values for each parameter are given.

From these simulation data it appears that upregulated ECMPs reached steady state values in shorter time, and that aggregation of receptors produced positive cooperativity. Considering the single divalent receptor, *C*_*d*_, the ECM:integrin binding pairs that had the highest steady state values include both collagen 1 and fibrinogen γ chain in association with the αvβ3 integrin receptor. The combinations with the shortest time to SS were collagen 1 binding αvβ3 or α1β1 receptors. For the aggregated receptor pairs, *A*_*dd*_, the pairs with the highest SS values include von Willebrand factor, fibrinogen γ chain, and collagen 1 binding αvβ3, as well as fibronectin binding α5β1. The ECM:integrin pairings with the shortest time to SS for *A*_*dd*_ pairs were collagen 1 binding αvβ3 and α1β1, and fibronectin binding α5β1. In nearly all cases, the CCl_4_ model ECM showed faster rise to SS compared to the control ECM; however, fibrinogen γ chain and Col 4α2 behaved in an opposite manner, owing to the fact that CCl_4_ actually downregulated these ECMPs in our dataset.

Sensitivity analysis was performed (Table 5) by evaluating the equilibrium constants listed in Table 3 for percent change in steady state after a ten-fold perturbation to the base values (Table 5). For simple integrin complex formation, i.e. when one ligand binds, increasing the K_i_ causes a significant increase in aggregated receptors, while a ten-fold decrease causes an approximate 100% decrease in aggregate pairing. Divalent receptors are moderately decreased when K_i_ increases, showing that decreased affinity suppresses the capacity for divalent receptor binding. Filling a single divalent receptor is negatively impacted by an increase in K_c_, with aggregate receptor pairs decreasing ~83% for all three receptor:ligand pairings. A decrease in Kc positively increases the steady state for aggregate pairs, because a lower K_eq_ for the second binding event increases affinity for receptors with one bound ligand. K_p_, the constant for filling empty paired receptors, was nominally affected by perturbation, as were perturbations to filling aggregate pairs. Finally, perturbations to the aggregation constant, K_a_, result in a decrease in steady sate for aggregated pairs when K_a_ is increased ten-fold, and an increase in steady state when K_a_ is decreased. This reflects the condition of a decreased equilibrium constant increasing the affinity for ligand binding, which is expected as the aggregation constant drives ligand affinity in this model.

**Table 5:** Sensitivity Analysis.

Parameter	Description	Baseline	10 fold increase	10 fold decrease	% Change in Steady State	
					10 fold increase	10 fold decrease
K_a_	**Aggregation Constant**					
avb3 (vWF)	Aggregate Receptor Pair	0.0014422	0.0001595	0.00731635	−88.9417	4.07317
	Single Divalent Receptor	0.047071	0.0496846	0.03206668	0.0555246	−31.8759
avb3 (Fn)	Aggregate Receptor Pair	0.0025167	0.0003024	0.00916465	−87.9828	2.64149
	Single Divalent Receptor	0.0448266	0.0494085	0.0250326	0.102215	−44.1568
a1b1 (Col I)	Aggregate Receptor Pair	7.787 × 10^−10^	7.788 × 10^−11^	7.7855 × 10^−9^	−89.9997	899.743
	Single Divalent Receptor	0.0001	0.0001	9.9988 × 10^−5^	1.4009 × 10^−5^	−0.0001401
K_p_	**Population of Empty Paired Receptors**					
avb3 (vWF)	Aggregate Receptor Pair	0.0014422	0.0012618	0.00144284	−12.5058	0.0466698
	Single Divalent Receptor	0.047071	0.0470725	0.04706991	0.00319051	−0.0022229
avb3 (Fn)	Aggregate Receptor Pair	0.0025167	0.0022089	0.00252032	−12.2318	0.142716
	Single Divalent Receptor	0.0448266	0.0448329	0.04482091	0.0140776	−0.0126299
a1b1 (Col I)	Aggregate Receptor Pair	7.787 × 10^−10^	7.489 × 10^−10^	7.7875 × 10^−10^	−3.83664	−4.768 × 10^−5^
	Single Divalent Receptor	0.0001	0.0001	0.0001	−2.454 × 10^−8^	−1.34 × 10^−10^
K_x_	**Aggregate Receptor Saturation**					
avb3 (vWF)	Aggregate Receptor Pair	0.0014422	0.0010941	0.00144273	−24.1374	0.03937633
	Single Divalent Receptor	0.047071	0.0470717	0.04707014	0.00154618	−0.0017387
avb3 (Fn)	Aggregate Receptor Pair	0.0025167	0.0019215	0.00251985	−23.650441	0.12406835
	Single Divalent Receptor	0.0448266	0.0448308	0.04482188	0.00949427	−0.0104646
a1b1 (Col I)	Aggregate Receptor Pair	7.787 × 10^−10^	7.06 × 10^−10^	7.7875 × 10^−10^	−9.3388012	−7.855 × 10^−5^
	Single Divalent Receptor	0.0001	0.0001	0.0001	−4.37 × 10^−9^	−2.26 × 10^−10^
K_i_	**Integrin Complex Formation**					
avb3 (vWF)	Aggregate Receptor Pair	0.0014422	0.0142873	2.8307 × 10^−5^	890.686233	−98.03722
	Single Divalent Receptor	0.047071	0.0306523	0.04969114	−34.880649	5.56644996
avb3 (Fn)	Aggregate Receptor Pair	0.0025167	0.0206362	5.2505 × 10^−5^	719.962077	−97.91377
	Single Divalent Receptor	0.0448266	0.0236912	0.04943082	−47.14931	10.2712401
a1b1 (Col I)	Aggregate Receptor Pair	7.787 × 10^−10^	9.496 × 10^−9^	1.3021 × 10^−11^	1119.42405	−98.327913
	Single Divalent Receptor	0.0001	9.694 × 10^−5^	1 × 10^−4^	−3.0666187	−0.0017795
K_c_	**Single Divalent Receptor Saturation**					
avb3 (vWF)	Aggregate Receptor Pair	0.0014422	0.0002512	0.00269898	−82.584496	87.1478824
	Single Divalent Receptor	0.047071	0.0426039	0.04702548	−9.4901208	−0.0966041
avb3 (Fn)	Aggregate Receptor Pair	0.0025167	0.0004241	0.00482601	−83.148576	91.7572123
	Single Divalent Receptor	0.0448266	0.0410334	0.04467362	−8.4619004	−0.3412082
a1b1 (Col I)	Aggregate Receptor Pair	7.787 × 10^−10^	1.279 × 10^−10^	1.2887 × 10^−9^	−83.571112	65.4777672
	Single Divalent Receptor	0.0001	9.695 × 10^−5^	9.9998 × 10^−5^	−3.0532152	−0.003242

Sensitivity Analysis for Integrin Receptor Concentration. Sensitivity analysis was performed by modulating equilibrium binding constants, calculating the percent change in steady state value attained by the system. Values for CCl_4_ treatment groups used for analysis.

## Discussion

Integrin binding to ECM is a vital mechanism for cell migration, invasion, proliferation, and signal transduction between cells and their microenvironment. Diseases of chronic inflammation and injury, including fibroses and cancer, involve persistent dysregulation of ECM-integrin processes and induce remodeling of the ECM. In addition to their intrinsic utility in cellular processes, association between immune cells and the ECM is regulated via the β1 & β3 integrin receptor subfamilies^29^. Elucidating these complex cell-ECM-driven pathological conditions could lead to improved prognostics and clinical outcomes via more precise therapeutic management of the tissue microenvironment. Several mathematical models of integrin binding have been reported with outputs relating to spatial clustering and signal transduction, liver fibrosis, and cell migration^17,19,30,31^. These models recapitulated certain aspects of integrin interactions; however, these previous studies typically modeled only one ligand, mainly fibronectin or collagen, and utilized generic cognate receptor.

In this study, the relative abundance of ECM components that are canonical substrates of integrin receptors was developed for the proposed modeling framework based on experimentally-obtained liver ECM data. With binding parameters from published literature, an integrin binding pattern was established for each integrin involved in hepatic processes that are involved in fibrosis. The model from Wanant *et al*. was adapted to implement the basic model for divalent binding^28^. Specifically, this model aptly describes initial integrin binding leading to a conformational switch of the receptor complex from low- to high-affinity. A model of receptor aggregation, which can describe integrin clustering upon attachment to ECM via adhesions^5^, was also implemented. The simulations include how each integrin binds with cognate ECM ligands and incorporates the varying affinities that drive this interaction. From these calculations, the kinetic indices of each integrin for each of its binding partners were determined separately. The impact of changes to the ECM (e.g., in response to CCl_4_-induced fibrosis) on integrin binding was modeled by calibrating the substrate concentration based on the proteomic analyses. The extracellular matrix proteome was consistent with the known disease phenotype of the mouse model, with upregulation of specific ECMPs involved in fulminant fibrosis. The computational results show that in simulations using these ECMPs as substrate for key integrin receptors, interactions involving profibrotic integrins were predominant.

The CCl_4_ mouse model of liver fibrosis was chosen here due to its robustly characterized pathology and ECM/integrin phenotype (Fig. 2). This model is imperfect in its recapitulation of human liver fibrosis, but it is the current research standard and therefore has well-defined pathology and changes to the ECM^32^. Using proteomic data from CCl_4_-exposed mouse livers, integrin binding can be explored within the context of fulminant fibrosis. Collagen type Iα1, type III and type IV are excessively deposited due to activated hepatic stellate cells (HSCs) in response to myofibroblastic transformation induced by activated Kupffer cells and damaged hepatocytes^33,34^. In agreement with these established phenomena, collagens I, III, and V were upregulated in the CCl_4_ cohort in the current study (Table 1). Collagen I is aberrantly produced in this mouse model, and collagen V, a potent nucleating effector for the co-upregulated fibronectin, exhibited a slight increase from trace levels. In contrast, collagen IV and XVIII levels were decreased relative to the control. Interestingly, collagen XVIII was identified at relatively minimal levels in the controls, and absent in the CCl_4_ treated animals (Table 1). This is contrary to an expected increase in collagen XVIII following CCl_4_ treatment^22^. Nevertheless, interactions simulated with this ECMP are still based on experimental proteomic analysis. Integrin receptors were not able to be resolved with this particular method of proteomic analysis, so further proteomic analysis of integrin adhesion complexes in culture is a key component of the future directions for this project.

Owing to their involvement in several critical functions that drive homeostasis and dyshomeostasis, integrins have been identified as key druggable targets in several diseases. For example, integrin inhibitors have been evaluated to suppress liver fibrogenesis, disrupt attachment and invasion of cancer cells, and to mediate immune response^35–38^. Regrettably, many of these drugs fail in early trials and rarely reach clinical use, perhaps due to an incomplete understanding of integrin binding kinetics, which are traditionally based on single-species models and assumptions; indeed, even antibodies and small peptide mimetics with specificities for multiple integrins have limited clinical application^39,40^. Though necessary to target multiple integrins to maximize efficacy *in vivo*, perhaps the missing link is knowing which targeted doses are most effective for each anti-integrin molecule. In attempting to begin to develop a predictive tool for effective dosing, the primary goal of this work was to create a framework to simulate simple receptor aggregation and reproduce positive cooperativity induced by aggregate pairing. The simulations were parameterized to analyze for positive cooperativity of binding in the divalent and aggregation cases. The steady state values and time to steady state for each pairing correlated to upregulation of key ECMPs in CCl_4_ liver injury (Fig. 5; Table 4). The integrin receptors that predominated simulations of occupancy were consistent with those known to be at play in the disease model (Fig. 2).

This study offers a first step in which the proposed modeling framework has been initially evaluated using data from a model of fulminant fibrosis and by which other liver pathologies and how the transitional remodeling of the ECM affects ECM-integrin interactions could be explored. We acknowledge that a more comprehensive test of the model and its assumptions would require further experiments, which will be pursued in follow-up work. By testing homeostatic conditions against experimental treatment models, this platform could be broadly employed to predict or confirm changes in integrin binding (and by extension, signaling) caused by remodeling of the hepatic ECM in response to insult or injury. Longer term, a more complex stochastic model for concurrent integrin binding building upon the results of this study could be developed that considers competitive binding of multiple species. This would lay the foundation for a more detailed and nuanced analysis of ECM:integrin interactions.

## Methods

All experiments were performed in accordance with the guidelines and regulations of the University of Louisville Office of Research Integrity and Institutional Review Board and Biosafety Committee.

### Animals and treatments

Male C57BL/6J mice (4–6 w) were purchased from Jackson Laboratory (Bar Harbor, ME). Mice were housed in a pathogen-free barrier facility accredited by the Association for Assessment and Accreditation of Laboratory Animal Care, and procedures were approved by the University of Louisville’s Institutional Animal Care and Use Committee. Food and tap water were provided ad libitum. Mice were administered CCl_4_ (1 ml/kg i.p.; diluted 1:4 in olive oil; Sigma-Aldrich, St. Louis, MO) 2×/wk for 4 wk. Twenty-four h after the last CCl_4_ administration, mice were anesthetized by injection of a ketamine HCl/xylazine solution (100/15 mg/kg i.m.; Sigma-Aldrich, St. Louis, MO). Other animals received the same dose of CCl_4_, but only once, and were sacrificed 12–72 h after intoxication. Blood was collected from the vena cava just prior to sacrifice by exsanguination and citrated plasma was stored at −80 °C for further analysis. Portions of liver tissue were frozen immediately in liquid nitrogen, while others were fixed in 10% neutral buffered formalin or embedded in frozen specimen medium (Tissue-Tek OCT compound, Sakura Finetek, Torrance, CA) for subsequent sectioning and mounting on microscope slides.

### 3-step ECM extraction

#### Sample preparation and wash

Snap-frozen liver tissue (75–100 mg) was immediately added to ice-cold phosphate-buffered saline (pH 7.4) wash buffer containing commercially available protease and phosphatase inhibitors (Sigma Aldrich) and 25 mM EDTA to inhibit proteinase and metalloproteinase activity, respectively. While immersed in wash buffer, liver tissue was diced into small fragments and washed five times to remove contaminants. Between washes, samples were pelleted by centrifugation at 10,000 × g for 5 min and wash buffer was decanted.

#### NaCl extraction

Diced samples were incubated in 10 volumes of 0.5 M NaCl buffer, containing 10 mM Tris HCl (pH 7.5), proteinase/phosphatase inhibitors, and 25 mM EDTA. The samples were gently mixed on a plate shaker (800 rpm) overnight at room temperature. The following day, the remaining tissue pieces were pelleted by centrifugation at 10,000 × g for 10 min. The supernatant was saved and labeled as the NaCl fraction.

#### SDS extraction

The pellet from the NaCl extraction was subsequently incubated in 10 volumes (based on original weight) of a 1% SDS solution, containing proteinase/phosphatase inhibitors and 25 mM EDTA. The samples were gently mixed on a plate shaker (800 rpm) overnight at room temperature. The following day, the remaining tissue pieces were pelleted by centrifugation at 10,000 × g for 10 min. The supernatant was saved and labeled as the SDS extract.

#### Guanidine HCl extraction

The pellet from the SDS extraction was incubated with five volumes (based on original weight) of a denaturing guanidine buffer containing 4 M guanidine HCl (pH 5.8), 50 mM sodium acetate, 25 mM EDTA, and proteinase/phosphatase inhibitors. The samples were vigorously mixed on a plate shaker at 1200 rpm for 48 h at room temperature; vigorous shaking is necessary at this step to aid in the mechanical disruption of ECM components. The remaining insoluble components were pelleted by centrifugation at 10,000 × g for 10 minutes. This insoluble pellet was retained and solubilized as described below. The supernatant was saved and labeled as the GnHCl fraction.

#### Deglycosylation and solubilization

The supernatants from each extraction were desalted using Zeba Spin columns (Pierce) according to manufacturer’s instructions. The desalted extracts were then mixed with five volumes of 100% acetone and stored at −20 °C overnight to precipitate proteins. The precipitated proteins were pelleted by centrifugation at 16,000× g for 45 min. Acetone was evaporated by vacuum drying in a RotoVap for one hour. Dried protein pellets were resuspended in 500 µL deglycosylation buffer (150 mM NaCl, 50 mM sodium acetate, pH 6.8, 10 mM EDTA, and proteinase/phosphatase inhibitors) that contained chondroitinase ABC (*P*. *vulgaris*; 0.025 U/sample), endo-beta-galactosidase (*B*. *fragilis*; 0.01 U/sample) and heparitinase II (*F*. *heparinum*; 0.025 U/sample). Samples were incubated overnight at 37 °C; those containing the pellet remaining after the guanidine HCl step received 20 µL DMSO for solubilization. Protein concentrations were estimated by absorbance at 280 nm using bovine serum albumin (BSA) in deglycosylation buffer for reference standards.

### LC-MS/MS analysis of samples

#### Sample cleanup and preparation for liquid chromatography

Pooled samples in deglycosylation buffer were thawed to room temperature and clarified by centrifugation at 5,000 × g for 5 min at 4 °C. Samples were reduced by adding 1 M DTT to 50 µL (25 µg) of each sample and then incubating at 60 °C for 30 min before addition of 8 M urea in 0.1 M Tris-HCl (pH 8.5) was added to each sample. Each reduced and diluted sample was digested with a modified Filter-Aided Sample Preparation (FASP) method. Recovered material was dried in a SpeedVac and redissolved in 200 µL of 2% v/v acetonitrile (ACN)/0.4% formic acid (FA). The samples were then trap-cleaned with a C18 PROTO^TM^ 300 Å Ultra MicroSpin Column (The Nest Group). The sample eluates were incubated at −80 °C for 30 min, dried in a SpeedVac, and stored at −80 °C. Before liquid chromatography, dried samples were warmed to room temperature and dissolved in 2%v/v ACN/0.1% FA to a final concentration of 0.25 µg/µL. A volume of 16 µL (4 µg) of sample was injected into the Orbitrap Elite.

#### Liquid Chromatography

Dionex Acclaim PepMap 100, 75 µM × 2 cm nanoViper (C18, 3 µm, 100 Å) trap and Dionex Acclaim PepMap RSLC, 50 µM × 15 cm nanoViper (C18, 2 µm, 100 Å) separating column were used. An EASY n-LC (Thermo) UHPLC system was used with mobile phase buffer A (2% v/v acetonitrile/0.1% v/v formic acid), and buffer B (80% v/v acetonitrile/0.1% v/v formic acid). Following injection of the sample onto the trap, separation was accomplished with a 140 min linear gradient from 0% B to 50% B, followed by a 30 min linear gradient from 50% B to 95% B, and lastly a 10 min wash with 95% B. A 40-mm stainless-steel emitter (Thermo P/N ES542) was coupled to the outlet of the separating column. A Nanospray Flex source (Thermo) was used to position the end of the emitter near the ion transfer capillary of the mass spectrometer. The ion transfer capillary temperature of the mass spectrometer was set at 225 °C, and the spray voltage was set at 1.6 kV.

#### Mass Spectroscopy

An Orbitrap Elite – ETD mass spectrometer (Thermo) was used to collect data from the LC eluate. An Nth Order Double Play with ETD Decision Tree method was created in Xcalibur v2.2. Scan event one of the method obtained an FTMS MS1 scan for the range 300–2000 m/z. Scan event two obtained ITMS MS2 scans on up to ten peaks that had a minimum signal threshold of 10,000 counts from scan event one. A decision tree was used to determine whether collision induced dissociation (CID) or electron transfer dissociation (ETD) activation was used. An ETD scan was triggered if any of the following held: an ion had charge state 3 and m/z less than 650, an ion had charge state 4 and m/z less than 900, an ion had charge state 5 and m/z less than 950, or an ion had charge state greater than 5; a CID scan was triggered in all other cases. The lock mass option was enabled (0% lock mass abundance) using the 371.101236 m/z polysiloxane peak as an internal calibrant.

#### Proteome Data Analysis

Proteome Discoverer v1.4.0.288 was used to analyze the data collected by the mass spectrometer. The database used in Mascot v2.4 and SequestHT searches was the 6/2/2014 version of the UniprotKB *Mus musculus* reference proteome canonical and isoform sequences. In order to estimate the false discovery rate, a Target Decoy PSM Validator node was included in the Proteome Discoverer workflow. The Proteome Discoverer analysis workflow allows for extraction of MS2 scan data from the Xcalibur RAW file, separate searches of CID and ETD MS2 scans in Mascot and Sequest, and collection of the results into a single file (.msf extension). The resulting.msf files from Proteome Discoverer were loaded into Scaffold Q + S v4.3.2. Scaffold was used to calculate the false discovery rate using the Peptide and Protein Prophet algorithms. The results were annotated with mouse gene ontology information from the Gene Ontology Annotations Database.

### Computational Modeling

First is considered the divalent receptor model that corresponds to ECM ligand binding of the α subunit occurring prior to the β subunit^7,28,41^, where *k*_1_ is the first-order association rate constant and *k*_2_ is the dissociation constant for singly occupied receptors (*C*_*m*_). *C*_*d*_ indicates a fully occupied integrin receptor with two bound ECM ligands, and *k*_3_ and *k*_4_ define the rate constants for association and dissociation, respectively, of the doubly bound integrin receptor. Differential equations for this model are:

1 $$\frac{dI}{dt}={k}_{2}{C}_{m}-{k}_{1}IE,$$

2 $$\frac{dE}{dt}={k}_{2}{C}_{m}-{k}_{1}IE+{k}_{4}{C}_{d}-{k}_{3}{C}_{m}E,$$

3 $$\frac{d{C}_{m}}{dt}={k}_{1}IE-{k}_{2}{C}_{m}+{k}_{4}{C}_{d}-{k}_{3}{C}_{m}E,$$

4 $$\frac{d{C}_{d}}{dt}={k}_{3}{C}_{m}E-{k}_{4}{C}_{d},$$

**Figure 6:**
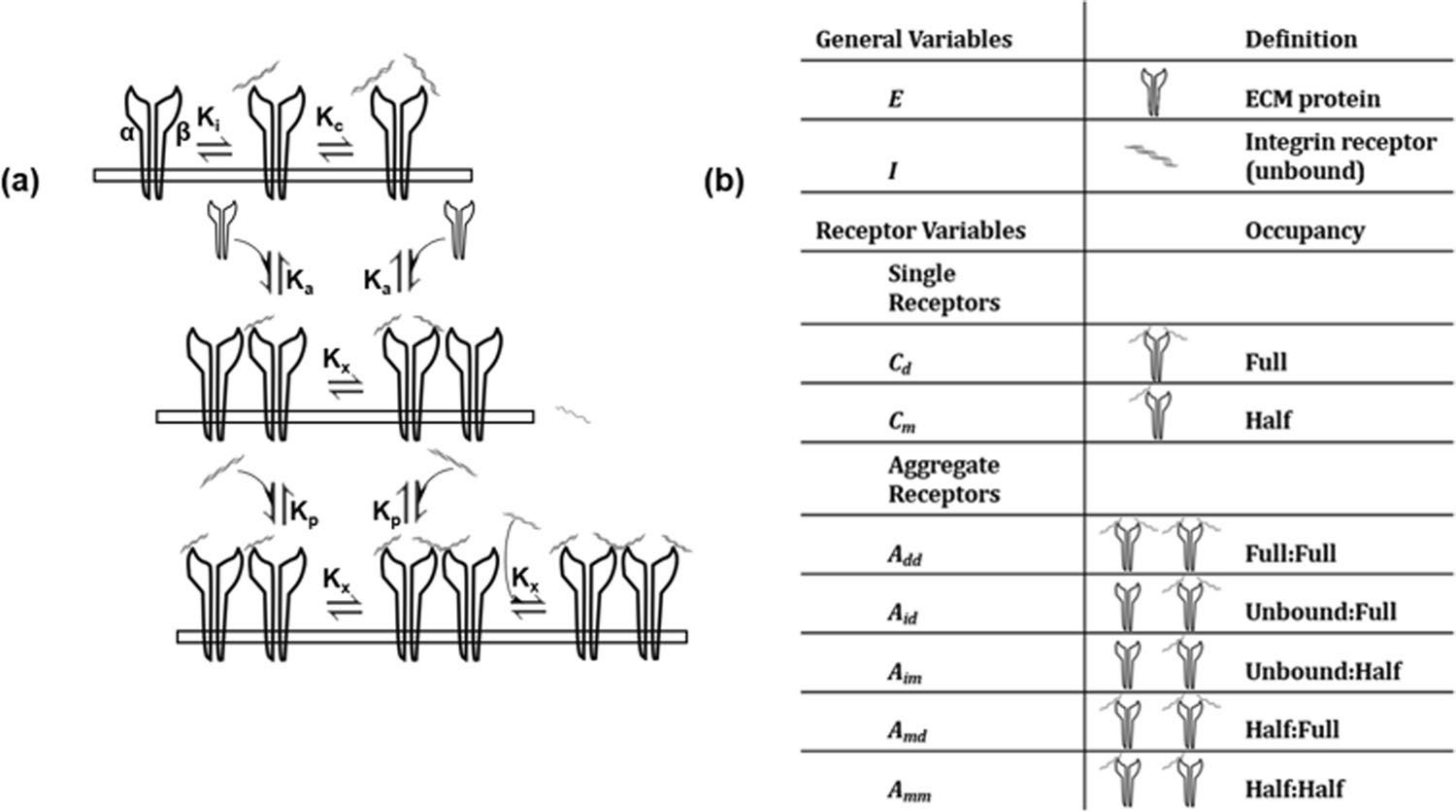
Model description: **(a)**, mass-action kinetics scheme of species variables. Divalent receptors bind ligand sequentially to α, β subunits, with K_i_ = *k*_*2*_ */k*_1_ for equilibrium of initial binding event (*C*_*m*_) and K_c_ = *k*_4_ */k*_3_ for fully occupied ECMP-Integrin receptor complex (*C*_*d*_). The receptor aggregation scheme incorporates divalent binding and receptor pairing such that half/fully occupied receptors can aggregate only with an unbound receptor [aggregation equilibrium constant K_a_ = *k*_6_ */k*_5_]. Binding constants for an additional ECM ligand binding to unbound receptor in an aggregate pair are the same regardless of occupancy status of its paired receptor [population equilibrium constant is K_p_ = *k*_10_ */k*_9_]. The equilibrium constant for adding a second ECM ligand to a singly bound receptor in any pair-configuration is K_x_ = *k*_8_ */k*_7_. Adapted from Wanant and Quon (2000)^28^. **(b)**, model species description.

The scheme for receptor aggregation and ligand binding is shown in Fig. 6. In the model of receptor aggregation, we utilized the same scheme as Wanant *et al*.^28^, wherein receptors pair in a manner such that either singly- or doubly-bound receptors can aggregate only with an unbound receptor with the aggregation equilibrium constant K_A_ (where K_A_ = *k*_5_ */k*_6_), and disaggregation equilibrium constant K_A’_ (K_A’_ = *k*_6_ */k*_5_). Binding constants for an additional ECM ligand binding to the unbound portion of an aggregate pair are the same regardless of whether the bound portion has one or two ligands, where the equilibrium association constant is K_C_ = *k*_9_ */k*_10_. The equilibrium constant for adding a second ECM ligand to a singly bound receptor in any pair-configuration is K_F_ = *k*_7_ */k*_8_. The differential equations describing receptor aggregation are listed below:

5 $$\frac{dI}{dt}={k}_{2}{C}_{m}-{k}_{1}IE+{k}_{6}({A}_{im}+{A}_{id})-{k}_{5}I({C}_{m}+{C}_{d}),$$

6 $$\frac{dE}{dt}={k}_{2}{C}_{m}-{k}_{1}IE+{k}_{4}{C}_{d}-{k}_{3}{C}_{m}E+{k}_{10}({A}_{mm}+{A}_{md})-{k}_{9}E({A}_{im}+{A}_{id})+\,{k}_{8}({A}_{id}+{A}_{md}+{A}_{dd})-{k}_{7}E({A}_{im}+{A}_{mm}+{A}_{md}),$$

7 $$\frac{d{C}_{m}}{dt}={k}_{1}IE-{k}_{2}{C}_{m}+{k}_{4}{C}_{d}-{k}_{3}{C}_{m}E+{k}_{6}{A}_{im}-{k}_{5}I{C}_{m},$$

8 $$\frac{d{C}_{d}}{dt}={k}_{3}{C}_{m}E-{k}_{4}{C}_{d}+{k}_{6}{A}_{id}-{k}_{5}I{C}_{d},$$

9 $$\frac{d{A}_{im}}{dt}={k}_{5}I{C}_{m}-{k}_{6}{A}_{im}+{k}_{10}{A}_{mm}-{k}_{9}E{A}_{im}+{k}_{8}{A}_{id}-{k}_{7}E{A}_{im},$$

10 $$\frac{d{A}_{id}}{dt}={k}_{5}I{C}_{d}-{k}_{6}{A}_{id}+{k}_{10}{A}_{md}-{k}_{9}E{A}_{id}+{k}_{7}E{A}_{im}-{k}_{8}{A}_{id},$$

11 $$\frac{d{A}_{mm}}{dt}={k}_{9}E{A}_{im}-{k}_{10}{A}_{mm}+{k}_{8}{A}_{md}-{k}_{7}E{A}_{mm},$$

12 $$\frac{d{A}_{md}}{dt}={k}_{9}E{A}_{id}-{k}_{10}{A}_{md}+{k}_{8}E({A}_{dd}-{A}_{md})+{k}_{7}E({A}_{mm}-{A}_{md}),$$

13 $$\frac{d{A}_{dd}}{dt}={k}_{7}E{A}_{md}-{k}_{8}{A}_{dd},$$

where *A*_im_ indicates an aggregate pair comprised of one unbound integrin receptor coupled with a singly-bound receptor; *A*_*id*_ is the same combination, except featuring a doubly-bound receptor. A pair with two singly bound receptors is defined as *A*_*mm*_, with two doubly bound receptors is *A*_*dd*_, and *A*_*md*_ indicates a singly bound receptor paired with a doubly bound one (Fig. 6).

The affinity of integrin receptors for ECM proteins fibronectin and laminin are generally in the micromolar range. The K_d_ measured for ECM:integrin and, in particular, fibronectin binding, ranges between approximately 10^−7^–10^−6^ M^42^; Takagi *et al*. report nanomolar K_d_ values for fibronectin binding^43^. Mallet *et al*. utilized a K_d_ of 2 × 10^−4^ M for tethered RGD peptides in their model of integrin binding^17^.

### Simulations

Computer simulations were run using Spyder for Tellurium software version 2.3.5.2; Python version 2.7^44^. Binding curves were plotted using SigmaPlot 13.0. The model was initialized using ligand concentrations from proteomic analysis (Table 1) and initial integrin concentrations were derived from published values. Ligand concentration was developed by collapsing the fractionated sample data using MudPIT functionality in Scaffold. Rappsilber *et al*. defined protein abundance index (PAI) for estimation of absolute protein abundance^45^, and Ishihama *et al*. report that the emPAI, i.e. exponentially modified PAI, is approximately proportional to protein abundance^25^. Using the emPAI quantitative method, proteomic output was normalized by the tissue loading concentration of 0.25 µg/µL; these values for concentration were then divided by the molecular weight of the protein to convert to molar concentration. Kinetic rates listed in Table 2 were used to calculate microrate parameters relative to the established binding rates from literature; where exact microrates were unavailable, rates were estimated from various published literature sources. Table 3 relates the equilibrium constants of the system relative to initial integrin complex formation, such that subsequent binding and clustering steps produce cooperativity when simulated in these proportions. Sensitivity analysis was performed by varying levels of integrin receptor concentration in 10-fold increments, to explore binding when surface membrane integrin receptor expression is upregulated or downregulated as a consequence of disease state or in response to microenvironmental fluctuations.

### Data Availability

The datasets generated and/or analysed during the current study are available from the corresponding author on reasonable request.

## Electronic supplementary material

Supplementary Table S1

## References

[1] Hynes RO (2009). The extracellular matrix: not just pretty fibrils. Science.

[2] Massey VL (2017). The hepatic “matrisome” responds dynamically to injury: characterization of transitional changes to the extracellular matrix. Hepatology.

[3] Cox TR, Erler JT (2011). Remodeling and homeostasis of the extracellular matrix: implications for fibrotic diseases and cancer. Dis Model Mech.

[4] Poole, L. G. & Arteel, G. E. Transitional remodeling of the hepatic extracellular matrix in alcohol-induced liver injury. *BioMed Res Intl* **2016** (2016). 10.1155/2016/3162670 PMC5098054 27843941

[5] Humphries JD, Byron A, Humphries MJ (2006). Integrin ligands at a glance. J Cell Sci.

[6] Hynes RO (1987). Integrins: a family of cell surface receptors. Cell.

[7] Hynes RO (2002). Integrins. Cell.

[8] Patsenker E, Stickel F (2011). Role of integrins in fibrosing liver diseases. Am J Physiol Gastrointest Liver Physiol.

[9] Campbell ID, Humphries MJ (2011). Integrin structure, activation, and interactions. Cold Spring Harb Perspect Biol.

[10] Masson-Gadais B, Pierres A, Benoliel AM, Bongrand P, Lissitzky JC (1999). Integrin (alpha) and beta subunit contribution to the kinetic properties of (alpha)2beta1 collagen receptors on human keratinocytes analyzed under hydrodynamic conditions. J Cell Sci.

[11] Legate KR, Wickstrom SA, Fassler R (2009). Genetic and cell biological analysis of integrin outside-in signaling. Genes & Dev.

[12] Pouwels J, Nevo J, Pellinen T, Ylänne J, Ivaska J (2012). Negative regulators of integrin activity. J Cell Sci.

[13] Zhao T, Li Y, Dinner AR (2009). How focal adhesion size depends on integrin affinity. Langmuir.

[14] Plow EF, Haas TA, Zhang L, Loftus J, Smith JW (2000). Ligand binding to integrins. J Biological Chem.

[15] Sánchez-Cortés J, Mrksich M (2009). The platelet integrin aIIbb3 binds to the RGD and AGD motifs in fibrinogen. Chem Biol.

[16] Garratt AN, Humphries MJ (1995). Recent insights into ligand binding, activation and signalling by integrin adhesion receptors. Acta Anatomica.

[17] Mallet DG, Pettet GJ (2006). A mathematical model of integrin-mediated haptotactic cell migration. . Bull Math Biol.

[18] Dutta-Moscato J (2014). A multiscale agent-based in silico model of liver fibrosis progression. Front Bioeng Biotechnol.

[19] Welf ES, Naik UP, Ogunnaike BA (2012). A spatial model for integrin clustering as a result of feedback between integrin activation and integrin binding. Biophys J.

[20] Baiocchini A (2016). *E*xtracellular matrix molecular remodeling in human liver fibrosis evolution. PLoS One.

[21] Iredale JP, Thompson A, Henderson NC (2013). Extracellular matrix degradationin liver fibrosis: Biochemistry and regulation. Biochimica et Biophysica Acta Mol Basis Disease.

[22] Duncan MB (2013). *T*ype XVIII collagen is essential for survival during acute liver injury in mice. Dis Model Mech.

[23] Jiang Y, Liu J, Waalkes M, Kang YJ (2004). Changes in the gene expression associated with carbon tetrachloride-induced liver fibrosis persist after cessation of dosing in mice. Toxicological Sci.

[24] Wisniewski JR, Hein MY, Cox J, Mann M (2014). A “proteomic ruler” for protein copy number and concentration estimation without spike-in standards. Mol Cell Proteomics.

[25] Ishihama Y (2005). Exponentially modified protein abundance index (emPAI) for estimation of absolute protein amount in proteomics by th enumber of sequeced peptides per protein. Mol & Cellular Proteomics.

[26] Peerschke EI, Francis CW, Marder VJ (1986). Fibrinogen binding to human blood platelets: effect of gamma chain carboxyterminal structure and length. Blood.

[27] Ware S, Donahue JP, Hawiger J, Anderson WF (1999). Structure of the fibrinogen gamma-chain integrin binding and factor XIIIa cross-linking sites obtained through carrier protein driven crystallization. Protein Sci.

[28] Wanant S, Quon MJ (2000). Insulin receptor binding kinetics: Modeling and simulation studies. J Theor Biol.

[29] Bruck R (1997). *T*he use of synthetic analogues of Arg-Gly-Asp (RGD) and soluble receptor of tumor necrosis factor to prevent acute and chronic experimental liver injury. Yale J Biol Med.

[30] Caré BR, Soula HA (2011). Impact of receptor clustering on ligand binding. BMC Systems Biol.

[31] Nieto N, Lutolf MP (2011). Extracellular matrix bioengineering and systems biology approaches in liver disease. Systems Synthetic Biol.

[32] Delire B, Stärkel P, Leclercq I (2015). Animal models for fibrotic liver diseases: What we have, what we need, and what is under development. J Clin Translational Hepatol.

[33] Friedman SL (2000). Molecular Regulation of hepatic fibrosis, an integrated cellular response to tissue injury. J Biological Chem.

[34] Liedtke C (2013). *E*xperimental liver fibrosis research: update on animal models, legal issues and translational aspects. Fibrogenesis Tissue Repair.

[35] Rosenow F (2008). *I*ntegrins as antimetastatic targets of RGD-independent snake venom components in liver metastasis. Neoplasia.

[36] Shimaoka M, Springer TA (2003). Therapeutic antagonists and conformational regulation of integrin function. Nature Rev Drug Discovery.

[37] Henderson NC (2013). *T*argeting of av integrin identifies a core molecular pathway that regulates fibrosis in several organs. Nature Med.

[38] Agarwal SK (2014). Integrins and cadherins as therapeutic targets in fibrosis. Front Pharmacol.

[39] Millard M, Odde S, Neamati N (2011). Integrin targeted therapeutics. Theranostics.

[40] Goodman SL, Picard M (2012). Integrins as therapeutic targets. Trends Pharmacol Sci.

[41] Sauro, H. M. *Enzyme Kinetics for Systems Biology*, *2nd Ed*.(Ambrosius Publishing and Future Skill Software, Seattle, 2012).

[42] Buck CA, Horwitz AF (1987). Cell surface receptors for extracellular matrix molecules. Ann Rev Cell Biol.

[43] Takagi J, Strokovich K, Springer TA, Walz T (2003). Structure of integrin a5b1 in complex with fibronectin. EMBO J.

[44] Choi, K., Medley, J. K., König, M., Stocking, K. & Cannistra, C. Tellurium, version 2.3.5.2. Co*mputer Program* (2014).

[45] Rappsilber J, Ryder U, Lamond AI, Mann M (2002). Large-scale proteomic analysis of the human spliceosome. Mol & Cellular Proteomics.

[46] Kislinger T, Gramolini AO, MacLennan DH, Emili A (2005). Multidimensional protein identification technology (MudPIT): Technical overview of a profiling method optimized for the comprehensive proteomic investigation of normal and diseased heart tissue. Proteomics and Disease.

[47] Schirmer EC, Yates IJR, Gerace L (2003). MudPIT: A powerful proteomics tool for discovery. Discov Med.

[48] Kim JK (2005). A novel binding site in collagen type III for integrins a1b1 and a2b1. J Biological Chem.

[49] Kern A, Eble J, Golbik R, Kühn K (1993). Interaction of type IV collagen with the isolated integrins a1b1. Eur J Biochem.

[50] Denis C, Williams JA, Lu X, Meyer D, Baruch D (1993). Solid-phase von Willebrand factor contains a conformationally active RGD motif that mediates endothelial cell adhesion through the alpha v beta 3 receptor. Blood.

[51] Xu Y (2000). Multiple binding sites in collagen type I for the integrins a1b1 and a2b1. J Biological Chem.

