# Supplementary material for: Modeling the Kinetics of Integrin Receptor Binding to Hepatic Extracellular Matrix Proteins
Source: Sci Rep. 2017 Sep 29;7:12444. doi: 10.1038/s41598-017-12691-y (PMC5622105; doi:10.1038/s41598-017-12691-y)
Supplement: Supplementary file 1 — Supplementary Table S1 [file 41598_2017_12691_MOESM1_ESM.doc]

# **Modeling the Kinetics of Integrin Receptor Binding to Hepatic Extracellular Matrix Proteins**

(Supplemental Information)

Shanice V. Hudson^1,2^, Christine E. Dolin^1^, Lauren G. Poole^1^, Veronica L. Massey^1^, Daniel Wilkey^1^, Juliane I. Beier^1^, Michael L. Merchant^1^, Hermann B. Frieboes^1,2,3^*, Gavin E. Arteel^1,4^*

^1^Department of Pharmacology and Toxicology, University of Louisville, Louisville, KY 40202, USA

^2^Deptartment of Bioengineering, University of Louisville, KY 40208, USA

^3^James Graham Brown Cancer Center, University of Louisville, KY 40202, USA

^4^University of Louisville Alcohol Research Center, University of Louisville, KY, USA

*Joint senior authorship

**Corresponding Author:**

Gavin E. Arteel, Ph.D.

Department of Pharmacology and Toxicology

University of Louisville Health Sciences Center

Louisville, KY, 40292

Phone#: +1 (502) 852-5157

FAX#: +1 (502) 852-3242

[gavin.arteel@louisville.edu](mailto:gavin.arteel@louisville.edu)

**S1 Table. Hepatic Extracellular Matrix Proteome.**

| **Protein name** | **Accession Number** | **MW**  **(kDa)** | **ECM function** | **Ref** | **Fraction** | **Protein Abundance Value (Total Ion Current, TIC)** | |
| --- | --- | --- | --- | --- | --- | --- | --- |
|  |  |  |  |  |  | Con | CCl_4_ |
| Collagen alpha-1(I) chain | CO1A1 | 138 | Component of collagen fibrins | 1 | NaCl | 65046 | 45131 |
|  |  |  |  |  | SDS | 137040 | 233730 |
|  |  |  |  |  | GnHCl | 341530 | 979990 |
|  |  |  |  |  | Pellet | 6211200 | 1.15E+07 |
| Collagen alpha-1(III) chain | CO3A1 | 139 | Component of collagen fibrils | 1 | NaCl | ND | ND |
|  |  |  |  |  | SDS | 0 | 50898 |
|  |  |  |  |  |  |  |  |
|  |  |  |  |  | GnHCl | 10775 | 295860 |
|  |  |  |  |  | Pellet | 609480 | 1124900 |
| Collagen alpha-1(IV) chain | CO4A1 | 161 | Component of collagen network | 1 | NaCl | ND | ND |
|  |  |  |  |  | SDS | ND | ND |
|  |  |  |  |  | GnHCl | ND | ND |
|  |  |  |  |  | Pellet | 260700 | 293360 |
| Collagen alpha-1(V) chain | CO5A1 | 184 | Fibrillogenesis, crosslinking | 1 | NaCl | ND | ND |
|  |  |  |  |  | SDS | ND | ND |
|  |  |  |  |  | GnHCl | ND | ND |
|  |  |  |  |  | Pellet | 48958 | 74101 |
| Collagen alpha-1(XVIII) chain | E9QPX1 | 182 | Basement membrane endostatin precursor | 2 | NaCl | ND | ND |
|  |  |  |  |  | SDS | ND | ND |
|  |  |  |  |  | GnHCl | ND | ND |
|  |  |  |  |  | Pellet | 99351 | 75714 |
| Collagen alpha-2(I) chain | CO1A2 | 130 | Component of collagen fibrils (contains collage-like domain) | 1 | NaCl | ND | ND |
|  |  |  |  |  | SDS | 0 | 132530 |
|  |  |  |  |  | GnHCl | 39421 | 399300 |
|  |  |  |  |  | Pellet | 1624700 | 3856100 |
| Collagen alpha-2(IV) chain | CO4A2 | 167 | Component of collagen network | 1 | NaCl | ND | ND |
|  |  |  |  |  | SDS | ND | ND |
|  |  |  |  |  | GnHCl | ND | ND |
|  |  |  |  |  | Pellet | 879090 | 957710 |
| Collagen alpha-3(V) chain | Q9JLI2 | 172 | Component of collagen network | 1 | NaCl | ND | ND |
|  |  |  |  |  | SDS | ND | ND |
|  |  |  |  |  | GnHCl | ND | ND |
|  |  |  |  |  | Pellet | 11202 | 127310 |
| Dermatopontin | DERM | 24 | Regulates fibrin formation and activity | 3 | NaCl | ND | ND |
|  |  |  |  |  | SDS | ND | ND |
|  |  |  |  |  | GnHCl | ND | ND |
|  |  |  |  |  | Pellet | 48415 | 311060 |
| Dystroglycan | DAG1 | 97 | Matrix organization, basement membrane assembly | 4 | NaCl | 258740 | 190500 |
|  |  |  |  |  | SDS | ND | ND |
|  |  |  |  |  | GnHCl | ND | ND |
|  |  |  |  |  | Pellet | ND | ND |
| Fibrinogen beta chain | FIBB | 55 | Hemostasis, cell signaling | 5 | NaCl | 430270 | 105610 |
|  |  |  |  |  | SDS | ND | ND |
|  |  |  |  |  | GnHCl | ND | ND |
|  |  |  |  |  | Pellet | ND | ND |
| Fibrinogen gamma chain | FIBG | 49 | Hemostasis, fibrinogen polymerization | 6 | NaCl | 903790 | 263500 |
|  |  |  |  |  | SDS | ND | ND |
|  |  |  |  |  | GnHCl | ND | ND |
|  |  |  |  |  | Pellet | ND | ND |
| Fibronectin | FINC | 273 | Scaffolding, ECM organization, regulation of ECM-cell interactions | 7 | NaCl | 0 | 468050 |
|  |  |  |  |  | SDS | 0 | 155800 |
|  |  |  |  |  | GnHCl | ND | ND |
|  |  |  |  |  | Pellet | 606430 | 2090400 |
| Galectin-1 | LEG1 | 15 | Matrix crosslinking, matrix organization | 8 | NaCl | 0 | 142740 |
|  |  |  |  |  | SDS | 31327 | 86901 |
|  |  |  |  |  | GnHCl | ND | ND |
|  |  |  |  |  | Pellet | 48789 | 96508 |
| Galectin-3-binding protein | LG3BP | 64 | Cell-matrix adhesion | 9 | NaCl | ND | ND |
|  |  |  |  |  | SDS | 0 | 94674 |
|  |  |  |  |  | GnHCl | ND | ND |
|  |  |  |  |  | Pellet | ND | ND |
| Plasminogen | PLMN | 91 | Precursor of plasmin (serine protease) | 10 | NaCl | ND | ND |
|  |  |  |  |  | SDS | 0 | 143750 |
|  |  |  |  |  | GnHCl | ND | ND |
|  |  |  |  |  | Pellet | 31440 | 647750 |
| Vimentin | VIME | 54 | Microtubule formation | 11 | NaCl | ND | ND |
|  |  |  |  |  | SDS | 0 | 97412 |
|  |  |  |  |  | GnHCl | ND | ND |
|  |  |  |  |  | Pellet | 524190 | 612110 |
| von Willebrand factor A | VMA5A | 87 | Hemostasis | 12 | NaCl | ND | ND |
|  |  |  |  |  | SDS | ND | ND |
|  |  |  |  |  | GnHCl | ND | ND |
|  |  |  |  |  | Pellet | 247000 | 105000 |

**S1 Table. Hepatic Extracellular Matrix Proteome.** Full data set for CCl_4_ fibrosis model liver ECM proteomic analysis, obtained experimentally for fractionated ECM samples.

Reference List

1. Ricard-Blum S. (2011) The collagen family. Cold Spring Harb Perspect Biol, 3(1):a004978.

2. Duncan MB, Yang C, Tanjore H, Boyle PM, Keskin D, Sugimoto, Zeisberg M, Olsen BR, Kalluri R (2013) Type XVIII collagen is essential for survival during acute liver injury in mice. Dis Model Mech 6: 942-951.

3. Wu W, Okamoto O, Kato A, Matsuo N, Nomizu M, Yoshioka H et al. (2013) Dermatopontin regulates fibrin formation and its biological activity. J Invest Dermatol, 134(1):256-63.

4. Henry MD, Campbell KP. (1998) A role for dystroglycan in basement membrane assembly. Cell, 95(6):859-870.

5. Mosesson MW, Siebenlist KR, Meh DA. (2001). The structure and biological features of fibrinogen and fibrin. Ann N Y Acad Sci, 936:11-30.

6. Mosesson MW. (2003) Fibrinogen gamma chain functions. J Thromb Haemost, 1(2):231-238.

7. To WS, Midwood KS. (2011) Plasma and cellular fibronectin: distinct and independent functions during tissue repair. Fibrogenesis Tissue Repair, 4:21.

8. He J, Baum LG. (2006) Galectin interactions with extracellular matrix and effects on cellular function. Methods Enzymol, 417:247-256.

9. Sasaki T, Brakebusch C, Engel J, Timpl R. (1998) Mac-2 binding protein is a cell-adhesive protein of the extracellular matrix which self-assembles into ring-like structures and binds beta1 integrins, collagens and fibronectin. EMBO J, 17(6):1606-1613.

10. Andreasen PA, Egelund R, Petersen HH. (2000) The plasminogen activation system in tumor growth, invasion, and metastasis. Cell Mol Life Sci, 57(1):25-40.

11. Schoumacher M, Goldman RD, Louvard D, Vignjevic DM. (2010) Actin, microtubules, and vimentin intermediate filaments cooperate for elongation of invadopodia. J Cell Biol, 189(3):541-556.

12. Hassam MI, Saxena A, Ahmad F. (2012) Structure and function of von Willebrand factor. Blood Coagul Fibrinolysis, 23(1):11-22.
